# The role of dispositional mindfulness in the fear-avoidance model of pain

**DOI:** 10.1371/journal.pone.0280740

**Published:** 2023-01-27

**Authors:** Jenna M. Wilson, Ilana Haliwa, Jerin Lee, Natalie J. Shook

**Affiliations:** 1 Department of Anesthesiology, Perioperative and Pain Medicine, Brigham and Women’s Hospital, Boston, MA, United States of America; 2 Department of Psychology, Salve Regina University, Newport, RI, United States of America; 3 Department of Psychological Sciences, University of Connecticut, Storrs, CT, United States of America; 4 School of Nursing, University of Connecticut, Storrs, CT, United States of America; Lorestan University, ISLAMIC REPUBLIC OF IRAN

## Abstract

**Objective:**

The fear-avoidance model of pain posits that a painful stimulus is interpreted through pain catastrophizing, which leads to negative downstream cognitions, emotions, and behaviors that shape the experience of pain. As dispositional mindfulness is associated with less catastrophizing and pain, some researchers have suggested incorporating mindfulness into the fear-avoidance model. Across two studies, we empirically tested dispositional mindfulness as a stand-alone component within the fear-avoidance model of pain.

**Methods:**

Two independent, online cross-sectional surveys (*N*s = 362 and 580 U.S. adults) were conducted. Participants completed validated assessments of mindfulness, pain catastrophizing, fear of pain, pain vigilance, depression, pain intensity, and pain sensitivity. Using structural equation modeling, we tested the inclusion of dispositional mindfulness in the fear-avoidance model of pain. We proposed that greater mindfulness would be associated with less pain catastrophizing, which in turn would be associated with less fear of pain, leading to less depression, and then ultimately less pain intensity and pain sensitivity.

**Results:**

Across both studies, the fear-avoidance model of pain did not fit the data well, with or without mindfulness included. We found that a simplified model fit the data best (Study 1: χ^2^/df = 1.83; CFI = .981; RMSEA = .049, 90% CI [0.019, 0.076]; SRMR = 0.031; Study 2: χ^2^/df = 2.23; CFI = .976; RMSEA = .046, 90% CI [0.026, 0.067]; SRMR = .031), such that greater mindfulness was significantly associated with less pain catastrophizing and, in turn, lower levels of pain intensity and pain sensitivity.

**Conclusion:**

Our findings suggest that a simplified model, compared to the traditional fear-avoidance model, may partly explain the experience of pain among individuals without chronic pain. Future work should examine the temporal associations among these variables to inform the employment of future empirically supported interventions for pain management.

## Introduction

Pain is an unpleasant physical experience that can be debilitating and represents the leading contributor to the global burden of disease and non-fatal disease [1]. The experience of pain is subjective, largely influenced by psychological processes. Indeed, according to the fear-avoidance model of pain, pain catastrophizing affects the interpretation of an insult or injury and initiates a cascade of cognitions, emotions, and behaviors that shape the experience and management of pain [2, 3]. Though this model has been used to guide research over the past few decades, relatively little empirical work has tested the full model [4]. Furthermore, some researchers have recently suggested revising the fear-avoidance model of pain to incorporate dispositional mindfulness [5], a positive psychological process associated with lower levels of pain and better pain management [6]. Thus, across two independent studies, we tested whether adding dispositional mindfulness as a stand-alone component in the fear-avoidance model of pain would result in a coherent model, fitting empirical data.

### Fear-avoidance model of pain

The fear-avoidance model of pain is a cognitive model of pain [2, 3]. The model depicts a process by which cognitive responses to an injury or painful stimulus can influence the perception and expectation of pain, resultant functional disability, and potential for recurring pain [2]. Central to the fear avoidance model is pain catastrophizing (i.e., ruminating on, magnification of, and feeling helpless about pain), a cognitive style that is proposed to initiate a series of responses to a painful stimulus that facilitates the development of pain [2, 7]. Specifically, when a pain experience is interpreted through pain catastrophizing, there is an increased affective response to pain (i.e., greater fear of pain). This affective response is proposed to lead to greater avoidance of and hypervigilance toward pain-related activities, and thus greater depression, disuse of affected body parts, and functional disability, which results in a worsened pain experience. Thus, the interpretation of a painful experience through catastrophic cognitions is associated with a host of maladaptive downstream effects that exacerbate pain, including pain intensity and sensitivity. Few studies have tested the proposed theoretical fear-avoidance model in its entirety, but existing empirical evidence has generally supported pathways within the model [4, 8]. However, researchers have suggested refining and expanding the model to incorporate additional constructs that have been associated with pain [4].

### Dispositional mindfulness and the fear-avoidance model of pain

Dispositional mindfulness represents an individual’s trait-level tendency to non-judgmentally attend to the present moment and accept thoughts and feelings without fixating on any one thing [9]. Independent of intentional mindfulness practices (e.g., yoga), individuals inherently differ in the extent to which they are mindful on a daily basis. Several cross-sectional studies have demonstrated an inverse association between dispositional mindfulness and both self-reported and behavioral pain outcomes among adults with and without chronic pain [6, 10–13]. However, more research is needed to elucidate how or why mindfulness is related to pain.

Schütze et al. suggested that mindfulness may be a separate component in the fear-avoidance model [5]. Indeed, dispositional mindfulness is inversely associated with several key components in the fear-avoidance model of pain (e.g., pain catastrophizing, depression, functional disability) [5, 14–16]. Furthermore, Schütze et al. tested dispositional mindfulness as a moderator of the relation between pain catastrophizing and the experience of pain [5]. They found that dispositional mindfulness moderated the relation between pain catastrophizing and pain intensity, such that the effect of pain catastrophizing on pain intensity was greater for individuals with lower levels of dispositional mindfulness. However, this moderation was not tested while accounting for the other constructs in the fear-avoidance model (e.g., fear of pain, depression). Thus, while initial evidence suggests that dispositional mindfulness may play a role in the fear-avoidance model of pain, and that it may particularly be related to pain catastrophizing, no research to date has empirically tested the entirety of the fear-avoidance model of pain with the incorporation of dispositional mindfulness.

Instead of a moderating path in the fear-avoidance model of pain, dispositional mindfulness may better be conceptualized as an independent factor in the model. The ability to self-regulate one’s awareness without judgment may play a significant role in the early stage of the pain experience where the cognitive process begins, functioning as a precursor to pain catastrophizing [5]. One mechanism by which mindfulness has been proposed to confer its salutary effects is through the reduction of negatively biased cognitions, or cognitions in which negatively valenced stimuli are more salient than positively valenced or neutral stimuli (e.g., rumination, dysfunctional attitudes, worry) [17, 18]. Mindfulness allows for redirection of attention away from negatively valenced stimuli and more accurate reappraisal of cognition, including positive reappraisal of maladaptive cognitions [18]. As pain catastrophizing can be characterized as a negatively biased cognition, mindfulness may directly influence the tendency to engage in pain catastrophizing. That is, more mindful individuals may be less likely to fixate on past experiences of pain or worry about the likelihood of pain reoccurring. Thus, dispositional mindfulness may be better conceptualized as an independent factor preceding pain catastrophizing in the fear-avoidance model of pain.

The primary aim of the present research was to empirically test dispositional mindfulness as a key component in the fear-avoidance model of pain, specifically occurring prior to pain catastrophizing. We hypothesized that greater dispositional mindfulness would be associated with less pain catastrophizing, which in turn would be associated with less fear of pain, leading to less functional disability (i.e., depression), and ultimately less pain (i.e., pain intensity and pain sensitivity). Study materials and data can be found at osf.io/r4f5y.

## Study 1

The purpose of Study 1 was to determine whether placing mindfulness before pain catastrophizing in the fear-avoidance model would account for acceptable statistical model fit. For this initial investigation, we assessed several components of the fear-avoidance model (i.e., pain catastrophizing, fear of pain, depression, pain sensitivity, and pain intensity) in a sample of U.S. adults.

### Materials and method

#### Participants and procedure

Participants were 468 U.S. adults recruited via Amazon’s Mechanical Turk (MTurk), an online crowdsourcing platform that is widely used for accessing large national samples. Recommended practices for improving data quality in MTurk studies [19] resulted in the exclusion of 106 participants due to problematic response patterns (i.e., straight line responses to close-ended questions, nonsensical open-ended responses, duplicate IP addresses; see S1 File). All analyses were conducted with and without excluded participants. The pattern of results and significance did not differ. The final sample was comprised of 362 individuals (50.3% women; *M*_age_ = 39.35 years, *SD* = 12.24; *Mdn*_*income*_ = $40,000 to $59,000; 45% married). Participants were 69.1% White, 11.6% Black, 11.3% Asian, 2.5% Hispanic/Latinx, 0.3% Native American, 3.0% ‘Other’, and 2.7% did not report their race/ethnicity.

The study was approved by the principal investigator’s university’s Institutional Review Board (IRB). Participants provided electronic informed consent prior to participating in the study. Participants completed the measures in a random order, except for a measure of state affect which appeared first and the demographics which appeared last. All participants were compensated with $1.00.

#### Measures

*Dispositional mindfulness*. Participants completed two assessments of dispositional mindfulness. There is currently a lack of consensus on the conceptualization of dispositional mindfulness [20] and we wanted to ensure that our findings were not specific to one conceptualization.

The 15-item Mindful Attention Awareness Scale (MAAS) measured attention to and awareness of present moment experience in one’s daily life [21]. Participants indicated how frequently they experience each item (e.g., ‘I do jobs or tasks automatically, without being aware of what I’m doing’) on a scale from 1 (*almost always*) to 6 (*almost never*). A mean composite score was computed, and higher scores reflect greater dispositional mindfulness.

The 12-item Cognitive and Affective Mindfulness Scale–Revised (CAMS-R) was also used to measure cognitive and affective components of dispositional mindfulness [22]. Participants indicated how frequently they experience each item (e.g., ‘It is easy for me to concentrate on what I am doing’) on a scale from 1 (ra*rely/not at all*) to 4 (*almost always*). Appropriate items were reverse scored, and a sum score was computed. Higher scores reflect greater dispositional mindfulness.

*Pain catastrophizing*. The 13-item Pain Catastrophizing Scale (PCS) was used to measure catastrophic thinking associated with pain [23]. Participants indicated the extent to which they have certain thoughts or feelings when they experience pain (e.g., ‘I worry all the time about whether the pain will end’) on a scale from 0 (*not at all*) to 4 (*all the time*). A composite score is computed by summing all the items, such that higher scores indicate greater pain catastrophizing.

*Fear of pain*. The 9-item Fear of Pain Questionnaire-9 (FPQ-9) measured fear associated with pain [24]. Participants indicated the extent to which they are fearful of experiencing the pain associated with each item (e.g., ‘Breaking your arm’) on a scale from 1 (*not at all*) to 5 (*extreme*). A sum score was computed, such that higher scores reflect greater fear of pain.

*Depression*. The 7-item depression subscale of the Depression Anxiety Stress Scales (DASS-21) was used as an indicator of functional disability [25]. Participants indicated how frequently they experienced items associated with depression (e.g., ‘I felt that life was meaningless’) over the past week on a scale from 0 (*did not apply to me at all*) to 3 (*applied to me very much or most of the time*). Scores across the items were summed, and higher scores reflect greater depressive symptoms.

*Pain sensitivity*. The 17-item Pain Sensitivity Questionnaire (PSQ) measured participants’ sensitivity to pain [26]. Participants are asked to imagine themselves in a series of situations (e.g., ‘Imagine you bump your shin badly on a hard edge, for example, on the edge of a glass table. How painful would that be for you?’) and indicate the extent to which each situation would be painful on a scale from 0 (*not at all painful*) to 10 (*most severe pain imaginable*). A mean score was computed excluding three filler items (e.g., ‘Imagine you take a shower with lukewarm water’), such that higher scores reflect greater pain sensitivity.

*Pain intensity*. The Brief Pain Inventory (BPI) was used to assess general pain intensity [27]. Participants rated their pain at its worst, least, and on average over the past week on a scale from 0 (*no pain*) to 10 (*pain as bad as you can imagine*). A mean score was computed such that higher scores reflect greater pain intensity.

*State affect*. The 20-item Positive and Negative Affect Schedule (PANAS) was used to measures participants’ state affect [28]. There are two subscales, each consisting of ten items assessing for positive affect and negative affect. Participants indicated the degree to which they were experiencing positive (e.g., ‘excited’, ‘strong’) and negative (e.g., ‘distressed’, ‘scared’) affective states in the present moment on a scale from 1 (*very slightly or not at all*) to 5 (*extremely*). This measure was included as a potential covariate. A composite score was created for each subscale by summing all appropriate items. Higher scores indicate greater positive and negative affect.

*Demographics*. Participants were asked about their age, gender, ethnicity/race, education, marital status, and family income. Because research has shown differences in pain processing based on participant characteristics, demographic variables were examined as potential covariates [29].

#### Data analyses

Based on a sensitivity analysis for a linear multiple regression with *R*^*2*^ increase, assuming α = .05 and power = .08, the sample size was large enough to detect a small-medium effect size (*f*^*2*^ = 0.05). Data were missing for ≤ 1% of the sample. Little’s MCAR test was not significant, *X*^2^(31, *N* = 362) = 29.42, *p* = .56, indicating that data in the sample were missing completely at random. Given the small percentage of missingness, no data imputation was used. We examined skewness and kurtosis to ensure all variables were normally distributed. Several variables (i.e., DASS-21, PCS, PSQ, BPI) violated assumptions of normality and were transformed using square root transformations. However, the pattern of findings remained the same when using transformed variables, and thus, data using non-transformed variables are reported.

Data were analyzed using structural equation models (SEM) in RStudio version 1.41106 with the lavaan package [30]. There were no violations of multicollinearity (VIFs ≤ 2.08, Tolerances ≥ 0.48). Path coefficients were calculated within SEM and are presented as standardized beta weights. The following commonly used goodness-of-fit indices were used to evaluate model fit: Comparative Fit Index (CFI) > .90, Root Mean Square Error of Approximation (RMSEA) < .08, and Standardized Root Mean Square Residual (SRMR) < .05 [31]. We performed an iterative process of removing individual model components to find the best fitting model. Based on prior studies comparing alternative models and recommendations, we relied on CFI difference to evaluate improvements in alternative models [32, 33]. Two models with a CFI difference of 0.010 suggests that the models are equivalent, and thus, a difference greater than 0.010 [33, 34] was interpreted as substantively important and indicated a significantly better model fit.

### Results

Descriptive statistics and Cronbach’s alpha for each measure, as well as bivariate correlations among all measures, are presented in Table 1. The measures of mindfulness (MAAS and CAMS-R) were positively associated. Greater dispositional mindfulness (MAAS and CAMS-R) was associated with less pain catastrophizing and lower levels of depression. Mindfulness was not significantly related to fear of pain, pain sensitivity, or pain intensity. Greater pain catastrophizing was associated with greater fear of pain, depression, pain sensitivity, and pain intensity. Greater fear of pain was associated with greater depression, pain sensitivity, and pain intensity. Higher levels of depression were associated with greater pain sensitivity and pain intensity. Greater pain sensitivity was associated with greater pain intensity.

**Table 1:** Descriptive statistics, Cronbach’s alphas, and bivariate correlations for Study 1 variables.

	1	2	3	4	5	6	7	8	9	10	11	12
1. CAMS-R	-											
2. MAAS	.46***	-										
3. Pain catastrophizing	-.23***	-.22***	-									
4. Fear of pain	-.06	-.02	.53***	-								
5. Depression	-.41***	-.32***	.52***	.33***	-							
6. Pain Sensitivity	.00	.04	.48***	.63***	.34***	-						
7. Pain Intensity	-.07	-.10	.54***	.41***	.43***	.53***	-					
8. PA	.42***	.28***	.10	.25***	-.09	.35***	.24***	-				
9. NA	-.20***	-.16***	.48***	.38***	.61***	.48***	.61***	.15**	-			
10. Age	.27***	.19***	-.08	-.10	-.22***	-.06	-.02	.14**	-.25***	-		
11. Race	-.06	-.10*	-.15**	-.20***	-.06	-.35***	-.19**	-.18***	-.20***	.16**	-	
12. Income	.01	.00	-.03	-.02	-.04	-.00	-.12***	.03	-.01	-.06	-.03	-
Mean	28.89	4.18	17.76	24.18	5.71	4.21	2.47	31.55	15.78	39.35	-	-
*SD*	6.01	1.07	13.26	7.96	5.99	2.04	2.41	9.68	8.94	12.24	-	-
α	.94	.88	.96	.89	.95	.96	.94	.93	.96	-	-	-

*Note*. CAMS-R = Cognitive and Affective Mindfulness Scale–Revised; MAAS = Mindful Attention Awareness Scale; PA = positive affect; NA = negative affect. Race (1 = white, 0 = not white).

*p < .05.

**p < .01,

***p < .001.

#### Mindfulness in the fear-avoidance model

**Fig 1:**
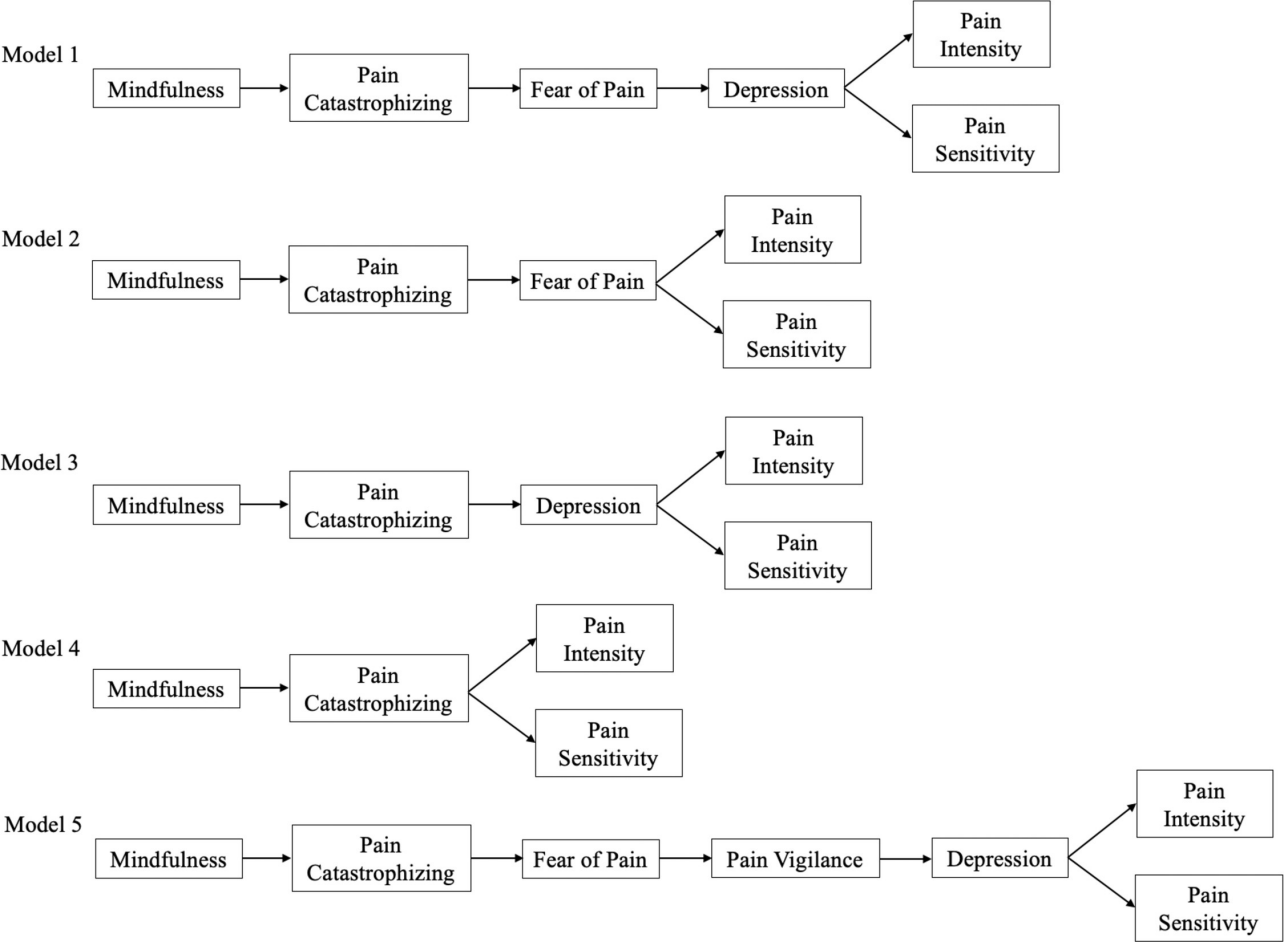
Structural equation models tested in Studies 1 and 2.

SEM was used to test the inclusion of dispositional mindfulness in the fear-avoidance model of pain. Our original model (see Model 1 in Fig 1) proposed that greater mindfulness would be associated with less pain catastrophizing, which in turn would be associated with less fear of pain, leading to less depression, and then ultimately less pain sensitivity and pain intensity. We controlled for state positive and negative affect, age, race, and income. All models were also estimated without covariates; the pattern of results remained the same when covariates were not included. A latent variable for mindfulness was created with the MAAS and CAMS-R.

Model 1 did not provide an adequate statistical fit to the data (χ^2^/df = 7.65; CFI = .824; RMSEA = .139, 90% CI [0.123, 0.156]; SRMR = .076), although all paths were significant. Greater dispositional mindfulness was significantly associated with less pain catastrophizing (β = -.15, 95% CI [-0.26, -0.04], *p* = .009) and pain catastrophizing was significantly associated with greater fear of pain (β = .44, 95% CI [0.34, 0.53], *p* < .001). Greater fear of pain was in turn associated with greater depression (β = .12, 95% CI [0.03, 0.21], *p* = .01). Greater depression was related to greater pain intensity (β = .15, 95% CI [0.05, 0.25], *p* = .004) and pain sensitivity (β = .18, 95% CI [0.08, 0.28], *p* = .001).

As the initial model did not fit well, we undertook an iterative approach of removing individual model components to determine whether model fit could be improved. For Model 2 (see Fig 1), depression was trimmed from the original model. As functional disability also encompasses limitations in performing activities of daily living [35], a measure of depression alone may not have been a sufficient conceptualization of this component of the fear-avoidance model. Model 2 provided a good statistical fit to the data (χ^2^/df = 3.04; CFI = .950; RMSEA = .077, 90% CI [0.057, 0.098]; SRMR = 0.38), and all paths were significant. Greater dispositional mindfulness was significantly associated with less pain catastrophizing (β = -.15, 95% CI [-0.26, -0.04], *p* = .009) and pain catastrophizing was significantly associated with greater fear of pain (β = .44, 95% CI [0.34, 0.53], *p* < .001). Greater fear of pain was in turn associated with greater pain intensity (β = .17, 95% CI [0.08, 0.26], *p* < .001) and pain sensitivity (β = .44, 95% CI [0.36, 0.52], *p* < .001). Based on the CFI difference, Model 2 fit the data significantly better than Model 1(**Δ** CFI = .126).

Although Model 2 provided a good statistical fit to the data, we tested Model 3 (see Fig 1) with fear of pain trimmed from the original model to determine whether simply removing a factor and simplifying the model accounted for fit improvement. Model 3 tested whether greater mindfulness is associated with less pain catastrophizing, which in turn is associated with less depression, and then ultimately less pain sensitivity and pain intensity. Model 3 did not provide a good statistical fit to the data (χ^2^/df = 5.53; CFI = .887; RMSEA = .115, 90% CI [0.096, 0.134]; SRMR = .060), but all paths were significant and in the expected direction. Greater dispositional mindfulness was significantly associated with less pain catastrophizing (β = -.15, 95% CI [-0.26, -0.04], *p* = .009) and pain catastrophizing was significantly associated with greater depression (β = .30, 95% CI [0.21, 0.39], *p* < .001). Greater depression was in turn associated with greater pain intensity (β = .15, 95% CI [0.05, 0.25], *p* = .004) and pain sensitivity (β = .18, 95% CI [0.08, 0.28], *p* = .001). Based on the CFI difference, Model 3 fit the data significantly better than Model 1 (**Δ** CFI = .063). However, the CFI difference between Model 2 and Model 3 suggested that Model 2 was a significantly better fitting model to the data (**Δ** CFI = .063).

Finally, we tested Model 4 (see Fig 1) with both fear of pain and depression trimmed from the original model. Model 4 tested whether greater mindfulness is associated with less pain catastrophizing, which in turn is related to less pain sensitivity and pain intensity. Model 4 provided a good statistical fit to the data (χ^2^/df = 1.83; CFI = .981; RMSEA = .049, 90% CI [0.019, 0.076]; SRMR = 0.031). Greater dispositional mindfulness was significantly associated with less pain catastrophizing (β = -.15, 95% CI [-0.26, -0.04], *p* = .009) and pain catastrophizing was significantly associated with greater pain intensity (β = .31, 95% CI [0.21, 0.42], *p* < .001) and pain sensitivity (β = .30, 95% CI [0.18, 0.42], *p* < .001). Based on the CFI difference, Model 4 fit the data significantly better than Model 1 (**Δ** CFI = .157). Additionally, the CFI difference between Model 2 and Model 4 suggested that Model 4 was a significantly better fitting model to the data (**Δ** CFI = .031).

The original fear-avoidance model of pain does not include mindfulness. Potentially, including mindfulness may have worsened the original model fit. Thus, we tested a model whereby greater pain catastrophizing is related to greater fear of pain, which in turn is related to greater depression, and then greater pain sensitivity and pain intensity. Indeed, greater pain catastrophizing was significantly associated with greater fear of pain (β = .44, 95% CI [0.34, 0.53], *p* < .001). Greater fear of pain was in turn associated with greater depression (β = .12, 95% CI [0.03, 0.21], *p* = .01). Greater depression was related to greater pain intensity (β = .15, 95% CI [0.05, 0.25], *p* = .004) and pain sensitivity (β = .18, 95% CI [0.08, 0.28], *p* = .001). However, this model did not provide a good statistical fit to the data (χ2/df = 11.91; CFI = .817; RMSEA = .178, 90% CI [0.156, 0.201]; SRMR = .072). Notably, this model provided a worse fit to the data relative to Model 1, although it was not a statistically significant difference (**Δ** CFI = .007).

### Discussion

Study 1 tested the inclusion of dispositional mindfulness in the fear-avoidance model. Overall, the proposed model did not adequately fit our data. The alternative model that best fit the data was one in which fear of pain and depression were removed, such that greater mindfulness was associated with less pain catastrophizing, and thus less pain sensitivity and pain intensity. These initial findings suggest that the association between dispositional mindfulness and the experience of pain may be accounted for simply by pain catastrophizing, rather than through pathways involving functional disability or fear of pain.

## Study 2

The purpose of Study 2 was to replicate and expand upon findings from Study 1. First, a larger, independent sample was recruited. Also, a measure of pain hypervigilance was added to fully assess all constructs proposed in the fear-avoidance model [2].

### Materials and method

#### Participants and procedure

Participants were 912 U.S. residents recruited via MTurk. Consistent with recommended practices for improving data quality in MTurk studies [19], 332 participants were excluded due to problematic response patterns (e.g., straight line responses to close-ended questions, nonsensical open-ended responses, duplicate IP addresses; see S1 File). All analyses were conducted with and without excluded participants. The pattern of results and significance did not differ. The final sample was comprised of 580 individuals (55.7% women; *M*_age_ = 38.13 years, *SD* = 12.23; *Mdn*_*income*_ = $40,000 to $59,000; 50.2% married). Participants were 75.3% White, 8.1% Black, 5.7% Asian, 5.7% Hispanic/Latinx, 3.4% ‘Other’, and 0.7% Native American.

The study was approved by the principal investigator’s university’s IRB. Participants provided electronic informed consent prior to participating in the study. Participants completed the measures in a random order, except for a measure of state affect which appeared first and the demographics which appeared last. All participants were compensated with $1.00.

#### Measures

Participants completed most of the measures described in Study 1. This included the Mindful Attention Awareness Scale (MAAS), Cognitive and Affective Mindfulness Scale–Revised (CAMS-R), Pain Catastrophizing Scale (PCS), Fear of Pain Questionnaire-9 (FPQ-9), Pain Sensitivity Questionnaire-9 (PSQ), Brief Pain Inventory (BPI), Positive and Negative Affect Schedule (PANAS), and demographic questions. Participants also completed the following additional measures.

*Depression*. Instead of the DASS which was used in Study 1, the abbreviated 9-item Patient Health Questionnaire (PHQ-9), consisting of the first 8 items, was used as an indicator of depression. The last item, which assesses for suicidal ideation, was excluded. Participants indicated how frequently they experienced symptoms (e.g., ‘Little interest or pleasure in doing things’) over the past week on a scale from 0 (*not at all*) to 3 (*nearly every day*). Items were summed, and higher scores reflect greater depressive symptoms.

*Pain vigilance*. The 16-item Pain Vigilance and Awareness Questionnaire (PVAQ) assessed attentional habits related to pain [36]. Participants indicated how frequently each item (e.g., ‘I am very sensitive to pain’) was true of their behavior over the past week on a scale from 0 (*never*) to 5 (*always*). Appropriate items were reverse scored, and a sum score was computed. Higher scores reflect greater attention to pain, or vigilance.

#### Data analyses

Based on a sensitivity analysis for a linear multiple regression with *R*^*2*^ increase, assuming α = .05 and power = .08, the sample size was large enough to detect a small effect size (*f*^*2*^ = 0.03). Data were missing for ≥ 36% of the sample on several measures due to a technical error with the survey administration software. Little’s MCAR test was not significant, *X*^2^(200, *N* = 580) = 208.76, *p* = .32, indicating that data in the sample were missing completely at random. Multiple imputation was used to estimate missing values. We examined skewness and kurtosis to ensure all variables were normally distributed. Several variables (i.e., MAAS, PHQ-9, PCS, PSQ, BPI) violated assumptions of normality and were transformed using square root transformations. However, the pattern of findings remained the same when using transformed variables, and thus, data using non-transformed variables are reported.

As in Study 1, data were analyzed using SEM in RStudio with the lavaan package [30]. There were no violations of multicollinearity (VIFs ≤2.21, Tolerances ≥0.45). Path coefficients were calculated within SEM and are presented as standardized beta weights. CFI (> .90), RMSEA (< .08), and SRMR (< .05) were used to examine model fit. A CFI difference of ≥ .010 was interpreted as better model fit [34].

### Results

Descriptive statistics and Cronbach’s alpha for each measure, as well as bivariate correlations among all measures, are presented in Table 2. The measures of mindfulness (MAAS and CAMS-R) were positively associated. Greater dispositional mindfulness (MAAS and CAMS-R) was associated with less pain catastrophizing, lower levels of depression, and less pain intensity. Mindfulness assessed by the MAAS was significantly related to less fear of pain, but mindfulness assessed by the CAMS-R was not significantly related to fear of pain. Additionally, neither measure of mindfulness was significantly related to pain vigilance or pain sensitivity. Greater pain catastrophizing was associated with greater fear of pain, pain vigilance, depression, pain sensitivity, and pain intensity. Greater fear of pain was associated with greater pain vigilance, depression, pain sensitivity, and pain intensity. Greater pain vigilance was associated with higher levels of depression and greater pain sensitivity and pain intensity. Higher levels of depression were associated with greater pain sensitivity and pain intensity. Greater pain sensitivity was associated with greater pain intensity.

**Table 2:** Descriptive statistics, Cronbach’s alphas, and bivariate correlations for Study 2 variables.

	1	2	3	4	5	6	7	8	9	10	11	12
1. CAMS-R	-											
2. MAAS	.42***	-										
3. Pain catastrophizing	-.31***	-.21***	-									
4. Fear of pain	-.07	-.08*	.48***	-								
5. Pain vigilance	-.08	-.00	.56***	.40***	-							
6. Depression	-.52***	-.33***	.54***	.25***	.23***	-						
7. Pain Sensitivity	-.07	-.07	.39***	.54***	.32***	.24***	-					
8. Pain Intensity	-.13**	-.12**	.49***	.25***	.33***	.46***	.28***	-				
9. PA	.40***	.13**	-.01	.17***	.08	-.17***	.30***	.07	-			
10. NA	-.30***	-.20***	.42***	.34***	.15***	.57***	.34***	.37***	.00	-		
11. Age	.15***	.15***	-.21***	-.18***	-.14**	-.28***	-.21***	-.03	.06	-.26***	-	
12. Race	.01	-.00	-.05	-.09*	-.04	-.03	-.25***	-.02	-.15***	-.08	.19***	-
13. Income	.13**	.07	-.11**	-.04	-.12**	-.23***	-.04	-.14**	.09*	-.13**	.10*	.06
Mean	28.82	4.08	16.05	23.08	39.52	6.35	3.94	2.47	31.15	14.61	-	-
*SD*	6.19	1.07	13.40	7.56	16.10	6.15	1.79	2.21	9.86	7.92	-	-
α	.89	.94	.97	.88	.90	.91	.95	.92	.93	.96	-	-

*Note*. CAMS-R = Cognitive and Affective Mindfulness Scale–Revised; MAAS = Mindful Attention Awareness Scale; PA–positive affect; NA = negative affect. Race (1 = white, 0 = not white).

*p < .05.

**p < .01,

***p < .001.

#### Mindfulness in the fear-avoidance model

First, the full fear-avoidance model of pain including mindfulness was tested (see Model 5 in Fig 1). The model proposed that greater mindfulness is associated with less pain catastrophizing, which in turn is related to less fear of pain, which leads to less pain vigilance and then less depression, and then ultimately less pain sensitivity and pain intensity. As in Study 1, we controlled for state positive and negative affect, age, race, and income. Models were also estimated without covariates and the pattern of results remained the same. A latent variable for mindfulness was created with the MAAS and CAMS-R.

Model 5 did not provide a good statistical fit to the data (χ^2^/df = 16.01; CFI = .717; RMSEA = .161, 90% CI [0.150, 0.173]; SRMR = .086), although all paths were significant. Greater dispositional mindfulness was significantly associated with less pain catastrophizing (β = -.19, 95% CI [-0.28, -0.11], *p* < .001) and pain catastrophizing was significantly associated with greater fear of pain (β = .41, 95% CI [0.34, 0.48], *p* < .001). Greater fear of pain was in turn associated with greater pain vigilance (β = .39, 95% CI [0.31, 0.46], *p* < .001) which was related to greater depression (β = .14, 95% CI [0.06, 0.21], *p* < .001). Greater depression was ultimately related to greater pain intensity (β = .35, 95% CI [0.27, 0.44], *p* < .001) and pain sensitivity (β = .13, 95% CI [0.03, 0.22], *p* = .004).

We then tested Model 4, which had the best statistical fit to the data in Study 1. Model 4 proposed that greater mindfulness is associated with less pain catastrophizing, which in turn is associated with less pain sensitivity and pain intensity. This model provided good statistical fit to the data (χ^2^/df = 2.23; CFI = .976; RMSEA = .046, 90% CI [0.026, 0.067]; SRMR = .031). Greater dispositional mindfulness was significantly associated with less pain catastrophizing (β = -.19, 95% CI [-0.28, -0.11], *p* < .001) and pain catastrophizing was significantly associated with greater pain intensity (β = .40, 95% CI [0.31, 0.49], *p* < .001) and pain sensitivity (β = .29, 95% CI [0.17, 0.40], *p* < .001). Based on the CFI difference, Model 4 fit the data significantly better than Model 5 (**Δ** CFI = .259). Model 4 also fit the data better than all other alternative models from Study 1, including the original fear-avoidance model of pain without mindfulness (see S1 File).

### Discussion

Study 2 replicated the findings of Study 1. Mindfulness added to the original fear-avoidance model did not provide the best fit to the data. The alternative model which best fit Study 2 data mirrored the best fitting model identified in Study 1, in which greater mindfulness was associated with less pain catastrophizing, and thus less pain sensitivity and pain intensity. The results of Study 2 provided additional evidence that greater dispositional mindfulness is linked to less pain through pain catastrophizing and suggests that the fear-avoidance model of pain should be simplified when mindfulness is included.

## General discussion

The present studies tested the inclusion of mindfulness in the fear-avoidance model of pain. Study 1 demonstrated that a simplified model consisting of pain catastrophizing as the mechanism linking dispositional mindfulness with the experience of pain best fit our data. Study 2 extended the preliminary findings by incorporating pain vigilance, an additional component in the fear-avoidance model, to better adhere to the original conceptualization of the model. However, results from Study 2 indicated that the simplified model (i.e., mindfulness, pain catastrophizing, and pain) still provided the best model fit. The original fear-avoidance model of pain, which does not include mindfulness, did not provide adequate fit within either of the present studies. These studies are the first to empirically test mindfulness as a component within the fear-avoidance model of pain, and results indicate that mindfulness may play a significant role in reducing pain through decreased pain catastrophizing.

Although prior cross-sectional research indicated a link between dispositional mindfulness and all constructs of the fear-avoidance model (i.e., pain catastrophizing, fear of pain, hypervigilance, functional disability, and pain intensity) [5], mindfulness was only consistently associated with pain catastrophizing and depression across both samples in the present studies. Thus, our findings provide initial evidence that dispositional mindfulness may be specifically linked to pain-related constructs through associations with pain-related cognition. This is consistent with existing work in the mental health literature which suggests that mindfulness may serve to reduce negatively biased cognitions [17, 18], and that the salutary effects of mindfulness on well-being may be conferred indirectly through reductions in maladaptive cognition [37, 38].

Indeed, based on prior research, we hypothesized that dispositional mindfulness would play a significant role in the fear-avoidance model through an inverse association with pain catastrophizing. This hypothesis was supported by the present findings. However, across both studies, the proposed models that more closely reflected the fear-avoidance model (i.e., including depression, pain vigilance, fear of pain) did not demonstrate adequate fit. Rather, results indicated that the best fitting model was a simplified one in which greater mindfulness is indirectly associated with less pain through less pain catastrophizing. Based on our findings, present oriented and non-judgmental awareness may influence the early stage of pain where the cognitive process begins, functioning as a precursor to pain catastrophizing.

Of note, prior work examining relations between mindfulness and the fear-avoidance model recruited individuals with chronic pain [5], whereas the present studies did not specifically target individuals with chronic pain. Although the fear-avoidance model is a cognitive model of chronic pain, we hypothesized that the process(es) underlying the association(s) between mindfulness and pain would hold across general samples as well with respect to their experiences of acute pain (i.e., brief pain experiences lasting ≤3 months) [39]. Mindfulness has been shown to affect pain not only in chronic pain samples, but also in healthy samples experiencing acute pain (e.g., thermal stimulation) [40]. However, our findings suggest that the constructs in the fear-avoidance model, such as avoidant behavior and functional disability, may not be necessary for understanding the development and maintenance of pain among non-chronic pain samples, particularly if dispositional mindfulness is included in the model. Although our findings are based on cross-sectional data, it is important to note that a few longitudinal studies have *not* found evidence of the temporal ordering of the constructs in the fear-avoidance model of pain among pain patients. For example, researchers failed to show that pain catastrophizing preceded pain-related fear and that pain-related fear preceded depression in individuals with musculoskeletal and HIV-related pain [41, 42]. Notably, prior empirical research has not tested the entirety of the fear-avoidance model. Therefore, longitudinal research examining the sequential ordering of all constructs within the model, with the inclusion of dispositional mindfulness, are necessary.

Given that dispositional mindfulness can be fostered through repeated cultivation of state mindfulness [43], our findings provide promising implications for the treatment and management of pain. Our results suggest that mindfulness may influence pain through pain catastrophizing, providing a viable point of early intervention in the management of pain. In other words, cultivating dispositional mindfulness may serve as a valuable protective factor when pain or injury occurs, and mindfulness-based interventions could help individuals nonjudgmentally attend to catastrophic appraisals of pain [44], thus attenuating pain catastrophizing and reducing downstream impacts of maladaptive pain-related cognition. Furthermore, the accessible nature of some mindfulness-based interventions (e.g., mobile applications, guided meditation scripts online) may allow individuals to take preventative measures to managing pain should pain or an injury arise. Although these findings need to be replicated within samples experiencing chronic pain, the present study provides initial evidence that mindfulness interventions may be effective tools to help with the management of acute pain experiences which do not meet the threshold for chronic pain.

These results should be considered in the context of some limitations. First, as previously mentioned, we did not recruit a chronic pain sample to test the present model. Thus, additional research is needed to replicate the present findings among individuals with chronic pain to determine whether the additional constructs of the fear-avoidance model (i.e., depression, vigilance, fear) may serve as more critical components in such a sample. Second, both studies relied on self-report measures, and future studies should seek to include other experimental measures of pain, such as using Quantitative Sensory Testing [45]. Third, we had a limited operationalization of the disuse, depression, and disability component of the fear-avoidance model. We only assessed depression and did not have measures of disuse or disability. As such, this component of the model was not fully conceptualized in our studies. However, no empirical study to date has tested the entirety of the fear-avoidance model, and future work is needed to comprehensively test this model both with and without mindfulness as a component. Finally, both studies were cross-sectional and correlational, preventing causal conclusions. However, researchers have shown that cross-sectional mediation analysis has the power to detect mediation while not inflating Type I error [46]. Experimental and longitudinal work is needed to further validate the associations between mindfulness, pain catastrophizing, and pain.

## Conclusions

Across two studies, the present research suggests that greater dispositional mindfulness is related to less pain catastrophizing, which in turn is linked with reduced pain experience. Findings suggest that this simplified model of pain, compared to the whole fear-avoidance model, may better explain the pain experience among individuals without chronic pain. As such, mindfulness interventions may provide useful tools for the management of pain experiences. More research is warranted to further examine the role of mindfulness in the fear-avoidance model of pain among individuals with and without chronic pain.

## Supporting information

S1 File (DOCX)
