# Supplementary material for: The role of dispositional mindfulness in the fear-avoidance model of pain
Source: PLoS One. 2023 Jan 27;18(1):e0280740. doi: 10.1371/journal.pone.0280740 (PMC9882899; doi:10.1371/journal.pone.0280740)
Supplement: S1 File — (DOCX) [file pone.0280740.s001.docx]

**Study 1**

**Results**

**Preliminary Analysis**

A series of independent t-tests and chi-square analyses were conducted to assess whether participants included in the present study differed from those who were excluded. The excluded respondents did not significantly differ from the final sample on gender, marital status, income, dispositional mindfulness, or fear of pain (*p*s > .05). Compared to the final sample, excluded participants were significantly younger (*M* = 36.09, *SD* = 11.61 vs. *M* = 39.35, *SD* = 12.24), composed of less White individuals (50% vs. 69.1%), and reported greater pain catastrophizing (*M* = 21.44, *SD* = 15.18 vs. *M* = 17.76, *SD* = 13.26), greater depressive symptoms (*M* = 7.93, *SD* = 6.52 vs. *M* = 5.71, *SD* = 5.99), greater pain sensitivity (*M* = 4.90, *SD* = 2.45 vs. *M* = 4.21, *SD* = 2.04), greater pain intensity (*M* = 3.59, *SD* = 2.74 vs. *M* = 2.47, *SD* = 2.41), greater positive affect (*M* = 34.41, *SD* = 9.31 vs. *M* = 31.55, *SD* = 9.68), and greater negative affect (*M* = 20.26, *SD* = 11.15 vs. *M* = 15.78, *SD* = 8.94; all *p*s< .05).

**Study 2**

**Results**

**Preliminary Analysis**

A series of independent t-tests and chi-square analyses were conducted to assess whether participants included in the present study differed from those who were excluded. The excluded respondents did not significantly differ from the final sample on marital status, income, or mindfulness as measured by the CAMS-R (*p*s > .05). Compared to the final sample, excluded participants were significantly younger (*M* = 34.56, *SD* = 10.50 vs. *M* = 38.35, *SD* = 12.38), composed of less women (26.5% vs. 55.7%), less White individuals (43.4% vs. 75.3%), and reported greater mindful as measured by the MAAS (*M* = 4.24, *SD* = 0.91 vs. *M* = 4.08, *SD* = 1.07), greater fear of pain (*M* = 28.76, *SD* = 8.77 vs. *M* = 23.18, *SD* = 7.61), greater pain catastrophizing (*M* = 26.88, *SD* = 13.75 vs. *M* = 15.86, *SD* = 13.22), greater pain sensitivity (*M* = 6.10, *SD* = 2.35 vs. *M* = 3.95, *SD* = 1.78), greater positive affect (*M* = 34.28, *SD* = 9.25 vs. *M* = 31.15, *SD* = 9.86), greater negative affect (*M* = 23.25, *SD* = 12.93 vs. *M* = 14.61, *SD* = 7.92), greater depressive symptoms (*M* = 10.80, *SD* = 7.00 vs. *M* = 6.23, *SD* = 6.08), greater pain vigilance (*M* = 46.07, *SD* = 15.59 vs. *M* = 39.08, *SD* = 16.33), and greater pain intensity (*M* = 4.54, *SD* = 2.95 vs. *M* = 2.49, *SD* = 2.14; all *p*s < .05).

**Model Testing**

A series of alternative models were conducted to reflect all models from Study 1. The Study 2 model proposed that greater mindfulness is associated with less pain catastrophizing, which in turn is related to less fear of pain, which leads to less pain vigilance and then less depression, and then ultimately less pain sensitivity and pain intensity

**Model 2**

For Model 2, depression and pain vigilance were trimmed from Model 1. This model did not provide a good statistical fit to the data (χ2/df = 6.21; CFI = .902; RMSEA = .095, 90% CI [0.080, 0.111]; SRMR = .044). Greater dispositional mindfulness was significantly associated with less pain catastrophizing (β = -.19, 95% CI [-0.28, -0.11], *p* < .001) and pain catastrophizing was significantly associated with greater fear of pain (β = .41, 95% CI [0.34, 0.48], *p* < .001). Greater fear of pain was in turn associated with greater pain intensity (β = .14, 95% CI [0.05, 0.23], *p* = .001) and pain sensitivity (β = .42, 95% CI [0.35, 0.49], *p* < .001).

**Model 3**

For Model 3, fear of pain and pain vigilance were trimmed from Model 1. This model did not provide a good statistical fit to the data (χ^2^/df = 10.81; CFI = .844; RMSEA = .130, 90% CI [0.115, 0.146]; SRMR = .057). Model 3 tested whether greater mindfulness is associated with less pain catastrophizing, which in turn is associated with less depression, and then ultimately less pain sensitivity and pain intensity. Greater dispositional mindfulness was significantly associated with less pain catastrophizing (β = -.19, 95% CI [-0.28, -0.11], *p* < .001) and pain catastrophizing was significantly associated with greater depression (β = .34, 95% CI [0.27, 0.40], *p* < .001). Greater depression was in turn associated with greater pain intensity (β = .35, 95% CI [0.27, 0.44], *p* < .001) and pain sensitivity (β = .13, 95% CI [0.04, 0.22], *p* = .005).

**Model 6**

For Model 6, depression was trimmed from Model 5. Model 6 tested whether greater mindfulness is associated with less pain catastrophizing, which in turn is related to less fear of pain, which leads to less pain vigilance, and then ultimately less pain sensitivity and pain intensity. This model did not provide a good statistical fit to the data (χ^2^/df = 12.21; CFI = .772; RMSEA = .139, 90% CI [0.126, 0.153]; SRMR = .067). Greater trait mindfulness was significantly associated with less pain catastrophizing (β = -.19, 95% CI [-0.28, -0.11], *p* < .001) and pain catastrophizing was significantly associated with greater fear of pain (β = .41, 95% CI [0.33, 0.48], *p* < .001), which in turn was significantly associated with greater pain vigilance (β = .39, 95% CI [0.31, 0.46], *p* < .001). Greater pain vigilance was then related to greater pain intensity (β = .27, 95% CI [0.10, 0.44], *p* < .001) and pain sensitivity (β = .24, 95% CI [0.03, 0.45], *p* < .001).

**Model 7**

For Model 7, fear of pain was trimmed from Model 5. Model 7tested whether greater mindfulness is associated with less pain catastrophizing, which in turn is related to less pain vigilance, which leads to less depression, and then ultimately less pain sensitivity and pain intensity. This model did not provide a good statistical fit to the data (χ^2^/df = 12.14; CFI = .802; RMSEA = .139, 90% CI [0.126, 0.152]; SRMR = .075). Greater trait mindfulness was significantly associated with less pain catastrophizing (β = -.19, 95% CI [-0.28, -0.11], *p* < .001) and pain catastrophizing was significantly associated with greater pain vigilance (β = .59, 95% CI [0.52, 0.65], *p* < .001), which in turn was significantly associated with greater depression (β = .13, 95% CI [0.06, 0.21], *p* < .001). Greater depression was then related to greater pain intensity (β = .35, 95% CI [0.27, 0.44], *p* < .001) and pain sensitivity (β = .13, 95% CI [0.04, 0.22], *p* = .004).

**Model 8**

For Model 8, fear of pain and depression were trimmed from Model 5. Model 8 tested whether greater mindfulness is associated with less pain catastrophizing, which in turn is related to less pain vigilance, and then ultimately less pain sensitivity and pain intensity. This model provided adequate statistical fit to the data (χ^2^/df = 5.46; CFI = .909; RMSEA = .088, 90% CI [0.073, 0.104]; SRMR = .044). Greater trait mindfulness was significantly associated with less pain catastrophizing (β = -.19, 95% CI [-0.28, -0.11], *p* < .001), which was then associated with greater pain vigilance (β = .59, 95% CI [0.52, 0.65], *p* < .001), and ultimately related to greater pain intensity (β = .27, 95% CI [0.12, 0.41], *p* < .001) and sensitivity (β = .24, 95% CI [0.07, 0.42], *p* < .001).

**Fear-Avoidance Model without Mindfulness**

We tested the original fear-avoidance model of pain without dispositional mindfulness. This model did not provide a good statistical fit to the data (χ2/df = 23.49; CFI = .709; RMSEA = .197, 90% CI [0.182, 0.213]; SRMR = .089). Notably, this model provided a significantly worse fit to the data relative to Model 4 (**Δ** CFI = .267).
